# Progress in Medicine

**Published:** 1899-04-08

**Authors:** 


### Progress in Medicine.

### FEVERS.

(*Continued from page* 9.)

**Typhoid.**—The Belfast epidemic has been reported on by J. Lorrain Smith,[1] who points out the two probable causes are (*a*) deficient drainage and cleansing, (*b*) possible infection of the water supply. He failed to obtain the typhoid bacillus from the water, but found the bacillus coli of virulent type. Now, many of these latter were "clumped" when serum from typhoid patients was added to them, 40 per cent. of the patients tested reacted to the bacillus coli, and not more than 60 or 70 per cent. to the bacillus typhosus of an active type when a high standard was taken. In consequence he argues that the bacillus coli is in some circumstances a cause of typhoid, and, secondly, that the water was certainly subject to fæcal contamination, for the bacilli coli here were of a definitely virulent type. In the Maidstone epidemic the water showed likewise the absence of a bacillus typhosus and the presence of a bacillus coli of this character. However, typhoid is endemic in Belfast, though the source of the late outbreak may probably be due to infection of the water through the excreta of some patients who lived on the catchment area. The Paisley epidemic was examined by Frankland,[2] who refused to decide absolutely as to its source, but noticed a most dangerous system of connection between the water mains and the sewers, and a large number of "dead ends" of the mains in the specially-affected area. The colon bacillus was also found in water from the taps of this area, and from four of the dead ends of mains. M. A. Veeder[3] asserts that the use of boiled water in the Spanish-American War failed to prevent the spread of typhoid till the agency of flies was taken into account, and guarded against. Infection by flies, as opposed to water-borne infection, produces crops of cases in one neighbourhood which have no common water supply, and follows the direction of the prevailing warm winds. It occurs chiefly where open closets are in use, and only during the summer. It may even arise where typhoid excreta have been buried in the earth without disinfection and the bacilli have come to the surface, or where flies have had access to them before disinfection. Volatile disinfectants, such as carbolic, should be replaced by more lasting ones, such as sulphate of copper, and disinfection must be carried out immediately the excreta are passed.

**Tropical Fevers.**—Mervyn Gordon[4] thinks that the bacillus of Malta fever, first described by Bruce, is allied to an alkali-producing type of bacillus coli. He also shows that it has flagella, from one to four in number, and the power of movement. He claims, by the way, similar motile powers and flagella for the plague bacillus. A commission is sitting in Bombay and taking evidence on the subject of the plague. Very little curative serum seems to be obtainable, and the results so far are of little practical value. Nuttal found that flies die after feeding on the bodies of persons dead from plague, but not for some days, so that they may become carriers of infection. Ogata, too,[5] discovered plague bacilli in fleas taken from the bodies of infected rats, and it has been further ascertained that the rats are not easily infected through the intestinal canal by feeding on excreta. Hence it seems probable that rats are infected by the fleas, and both are necessary for the spread of plague among human beings in cases where direct or water-borne contamination is impossible. At Poona 4,179 patients were treated in the plague hospital, of whom 2,836 died. In the report of this hospital E. L. Marsh[6] notices that the non-bubonic cases showed the greater mortality, and that these were usually those of a pneumonic or septic and malignant type. The chief aim of treatment was to relieve the stress on the circulatory system, where the effects of the toxin are seen most prominently. Galeotti's investigations showed that this poison is a nucleoproteid which leads to intravascular coagulation and consequent change of blood pressure; but, besides this, it paralyses the circulatory machinery in some way, but whether by acting on the nervous elements or on the muscular ones is not clear. Lewin[7] remarks that no other fever is marked by such profound exhaustion at its very commencement, though at the height of the malady the sense of weakness may disappear, and the patient may even get up. The curious thick white coating of the tongue and the swelling of the inguinal and preauricular glands, the inflammation of the lungs, and the psychical troubles with fixed ideas of a depressing type are among the most striking phenomena of the disease.

**Malaria.**—Italian investigators have again succeeded in producing malaria in a human being by means of mosquito bites.[8] The mosquitos were brought from an infected district and produced the benign tertian type of malaria in the person bitten by them. Bignami and Grassi[9] had obtained previously a similar result with the æstivo-autumnal fever, which was produced with the consent of a patient in the Santo Spirile Hospital by the bites of mosquitos brought from Maccarese, where that type of fever abounds. These observers regard three species of the insects as concerned in the propagation of malaria in Italy, viz., Anopheles claviger, Culex penicillaris, and an unidentified one, named provisionally Culex malariæ.[10] They traced the development of the semilunar bodies in the intestines of the first-named species for some days after a meal of malarial blood. The changes corresponded with those seen by Ross in the proteosoma or malarial plasmodium of birds. They hold that the gray mosquito which Ross noted as the conveyer of bird malaria is the Culex pipiens, and is not concerned in transferring human malaria. Indeed, it is found very often in non-malarial places. As to the question whether the disease is conveyed otherwise than by mosquitos, they argue that it is not an air-borne disease, for the microbes cannot bear any desiccation, and are never found in the bronchial secretion, and we also know that germs are hardly ever lifted by the air from moist surfaces, as the marshes where malaria flourishes. Manson[11] considers that the flagella of the plasmodes are really flagellated spores which make their way into the wall of the intestinal canal of the mosquito. Macallum has noticed that they penetrate certain plasmodial spheres, and after this impregnation the sphere, at least in bird malaria, becomes an actively moving body which apparently penetrates the stomach wall of the mosquito. Finally, these or some similar form burst and set free a vast number of rodlets which pass with the poison of the mosquito's sting or sucker into the body of the new host. Laveran[12] points out that we have still to determine some of the stages of this process, and to verify the theory that mosquitos convey infection to man as they do to birds. Koch and his fellow commissioners confirm the history of the development of the parasite in bird malaria, as discovered by Ross and Macallum, and mention the curious fact that the central parts of the city of Rome are free from malaria, though the food and water used come from highly malarious places in the environs. Malaria, however, is found in those parts of Rome where there are gardens in which mosquitos can breed. This is, of course, strongly against the water-borne theory of the disease. It should be noticed, however, that Laurie continues to attack the plasmodial origin of malaria, and again in his last paper[13] asserts that the Laveran bodies are merely degenerate blood cells, incapable of being grown outside the body. L. H. Warner[14] claims that he has reproduced these bodies in cultures in normal blood kept at a temperature of 100 deg., but his paper is extraordinarily obscure, though he seems to have started his cultures in some way from mosquitos.

The diagnosis of malaria from typhoid has been discussed by Dock, Gilman Thomson, and Ewing in the light of their experiences during the late war. G. Thomson[15] reports on 200 cases, seen in six weeks, in many of whom the clinical symptoms were doubtful. Blood examination, however, was of great value, and some 17 were found to be suffering from both diseases. In such instances the malaria usually remains quiescent till the typhoid is over, but occasionally chills occur during the active course of typhoid and may be controlled by quinine. The Widal test and the search for plasmodia are thus of high importance in doubtful cases, even though negative results from either cannot be taken to prove the absence of the corresponding microbe. Brannan, in view of the difficulty at times of finding the plasmodium, lays it down that a malarial case should never be discharged as cured till the hæmoglobin and number of red cells in the blood is normal, as well as the plasmodium undiscoverable. Ewing[16] from Camp Wikoff, after examining 800 cases, confesses that occasionally plasmodia cannot be detected in typical and fatal cases of æstivo-autumnal fever, though they may sometimes after death be found in the spleen. He explains this by the known tendency of the ring-shaped organisms to disappear from the blood in the third or fourth day of the paroxysm. Six hundred of his cases turned out malaria, the plasmodia being found in 335 instances besides 40 where typhoid occurred in malarial subjects.

[1] Lancet, Dec. 24. [2] Brit. Med. Jour., Dec. 17. [3] Med. Rec, Jan.. 7. [4] Lancet, Mar. 11. [5] Practitioner, Dec. [6] Glasgow Med. Jour., Jan. [7] Brit. Med. Jour., Dec. 10. [8] Brit. Med. Jour., Dec. 17. [9] Brit. Med. Jour., Nov. 12. [10] Brit. Med. Jour., Dec. 10. [11] Practitioner, Nov. [12] Brit. Med. Jour., Feb. 18. [13] Lancet, Dec. 3. [14] New York Med. Jour., Dec. 10. [15] Med. Rec., Nov. 19. [16] New York Med. Jour., Feb. 4.

### DISEASES OF THE HEART AND CIRCULATION.

**Pericarditis.**—Giraudeau[1] insists, in opposition to Brentano, that tapping should always be preferred to incision in cases of large pericardial effusions, and that incision should be used only when the former fails. This applies even to purulent effusions which are occasionally cured by tapping alone, the only exceptions being putrid effusions. If a trocar is used it is well to puncture the skin with a knife first; it should be inserted obliquely as nearly parallel to the surface of the heart as possible. The author always taps in the fourth or fifth intercostal space about two inches to the left of the sternum, though it is certain that the two layers of the left pleura are frequently perforated. Theoretically, pneumothorax might result, but has never been recorded, and though empyema or even pneumonia have been known to follow, they are rare accidents. The heart has been wounded, occasionally fatally, but this accident is unlikely if an exploring syringe is inserted before the trocar. If the fluid is purulent and re-collects after one tapping, incision and drainage are indicated. Whatever the nature of the fluid, the immediate object and result of tapping is to relieve the most urgent symptoms. Permanent cure may follow, as in rheumatic cases; but the cure is more apparent than real, adherent pericardium, fibroid mediastinitis, &c., being sequelæ.

Chimenti[2] records the case of a girl, aged 19, who was admitted with purulent pericarditis following influenza. On admission the girl was almost moribund, with a temperature of 104, and extreme dyspnœa. There was bulging of the precordial region, absence of the apex beat, and dulness extending from the left axillary line to the mid-clavicular line on the right, and from the second to the seventh left intercostal space. An exploratory puncture in the fourth space showing the presence of pus, a free incision was made (without any anæsthetic) and the pericardium opened, and more than a litre of thick, flocculent pus let out. The cavity was well washed out with warm, sterilised, boracic solution and drained. The after history was uneventual, and the patient left the hospital in 45 days cured. She was seen two months later, and had kept well.

Sequeira[3] records a case of indurative mediastinito-pericarditis in a young infant—a very rare condition, which has received but scanty recognition. An infant aged 15 months was admitted to hospital; on examination it was found that the area of cardiac dulness was enlarged upwards to the upper border of the second intercostal space, and also beyond the middle line of the sternum, except over the manubrium. The heart sounds were normal. The lungs were normal. The liver and spleen were not palpable below the ribs. There was no tenderness or rigidity of the abdomen, which contained no fluid. The urine was of s.g. 1,015, and contained no albumen. The legs pitted slightly on pressure. The child gradually got worse and died in two months, with all the symptoms of cardiac dilatation. At the post-mortem examination it was found that the mediastinal glands were enlarged with foci of caseation. The pericardium was about three-quarters of an inch thick, fibrous, adherent everywhere to the heart and to the anterior and posterior mediastinal tissues. The valves of the heart were healthy, and both ventricles were hypertrophied and dilated. The liver was "nutmeg," and the abdomen contained ascitic fluid. Such cases are of interest, clinically, because of the difficulty in diagnosing the condition, and pathologically, because their etiology is so obscure. In the present case, except an increased area of cardiac dulness and occasional lividity of the extremities, there was nothing for weeks to make one suspect anything wrong with the heart. Cases such as these differ, on the one hand, from those of obviously tuberculous pericarditis, with generally much other evidence of tubercle, and, on the other hand, from the obviously rheumatic cases where there is endocarditis as well.

**Pulmonary Regurgitation.**—Boyd[4] has recorded two cases of pulmonary regurgitation, the first due to endocarditis affecting the pulmonary valve, and the second due to increased pressure within the pulmonary circuit resulting in dilatation of the pulmonary artery. Pulmonary regurgitation, when not congenital, is due to either inflammation of the valve or to dilatation, as in the two cases described. Of the former class of case only about a fourth were rheumatic. The essential points in the diagnosis are the location of the point of maximum intensity in the bruit in the second inter-space to the left of the sternum, the conduction of the bruit down the left border of the sternum, the area of audition wide outwards and to the left and very limited upwards or to the right, and the absence of any characteristic alteration in the pulse as is found in aortic incompetence.

**Malignant Endocarditis.**—Herzog[5] gives details of nine cases of this disease, of which six occurred in men and three in women. The ages varied from 19 to 50. All the cases terminated fatally. The patients often gave a history of previous rheumatic pains. Swelling of the joints was often found, and even a purulent effusion. In two cases traumatism appeared to be the starting point of the infection. In one case the endocarditis occurred along with malaria. Often the cause of the disease was quite obscure, the patients being admitted with delirium or coma. The diagnosis from meningitis, uræmia, and typhoid fever may be difficult. Malignant endocarditis may be part of a general infection, of which a small wound may be the starting-point. It may also supervene upon other infective diseases. The symptoms are variable. Even the clinical evidence of a lesion of the valves may be slight, or even wanting, and the temperature may show nothing characteristic. Often a parenchymatous nephritis was present, and in one case an acute hæmorrhagic nephritis appeared during the course of the disease. In this case the malignant endocarditis followed an attack of acute rheumatism. In two cases hemiplegia occurred as the consequence of embolism.

**Toxæmic Delirium in Heart Disease.**—Eichhorst[6] draws attention to the fact that patients with uncompensated heart disease in whom diuresis is brought about by cardiac tonics often suffer from characteristic symptoms. Somnolence may first appear, and then delirium. At times the patient mumbles incomprehensible words and sentences, or shouts out, or becomes excited and violent. Thus, to the apathetic state there succeeds one in which there is much tossing about of the body. The pupils are small, and often unconsciousness may follow. The patient breathes deeply and hurriedly, but the face is not cyanosed. This condition may last several days, to cease when the œdema has disappeared and the polyuria due to the digitalis has come to an end. The condition is not serious, all the author's cases recovering. The symptoms cannot be looked upon as uræmic. Eichhorst believes that they are due to the absorption of some poison into the blood from the œdematous fluid which is not excreted quickly enough by the kidney. In this way the central nervous system is poisoned.

**Essential Paroxysmal Tachycardia.**—Laache[7] thinks that the disease may be looked up as a neurosis, but not infrequently it is associated with evident changes in the heart valves or myocardium. The attack begins suddenly as a rule. In spite of the increased frequency the pulse is regular. As a rule, the temperature is normal. The attack stops as suddenly as it began, after a duration which varies from a few minutes to hours or even days. The prognosis for a single attack are good, though recurrences will probably take place. The diagnosis is easy, as a rule. The neuralgic pains of angina, as well as the dyspnœa of asthma, are lacking. Abdominal pain without the existence of any symptoms of abdominal disease is very striking. Martins looks upon paroxysmal tachycardia as a very acute cardiac dilatation which disappears as suddenly as it began. Laache considers this dilatation as secondary, and seeks for a cause of the disease partly in a neurotic and partly in an anatomical change in the heart muscle and cardiac vessels. The treatment consists in absolute rest during the attack, and the application of an ice-bag. Subcutaneous injections of morphia tend to alleviate symptoms, but do not shorten the attack. Of the nervous sedatives, the salts of bromine are recommended. In organic disease digitalis is the sovereign remedy.

**Aortic Aneurysm.**—Short[8] has recently published details of a case of aortic aneurysm in which pressure symptoms were limited to the trachea. A patient, aged 32, was admitted complaining of an irritable cough and difficulty of breathing on exertion. On examination slight general bronchitis was found with very harsh breath-sounds. The heart apex was just outside the normal position; there was no cardiac murmur, and no dulness, pulsation, or murmur was detected in the usual aneurysmal areas. The pulses were equal and normal; both vocal cords moved perfectly. At the back of the chest on the right side, just internal to the angle of the scapula, a deep distant pulsation could be detected over a small area. The cough, though not ringing in character, was a little brassy, and had a peculiar break in it, like a turkey's gobble. Such a cough is encountered in three conditions—a foreign body in the trachea, ulceration of the trachea, and pressure on the trachea. A diagnosis of aneurysm was made on the following grounds: (1) The presence of pressure signs without venous fulness; (2) the enlargement of the left ventricle, without evidence of renal or valvular disease; (3) dyspnœa increased on exertion; (4) absence of glandular enlargement; (5) gradual emaciation; (6) certain evidence of previous syphilis; (7) the age; and (8) the peculiar cough. The patient gradually became worse, and died from gradual cardiac failure. After death a small saccular aneurysm, which might have held a walnut, was found arising from the upper part of the arch of the aorta, pressing directly on the trachea, though the tube was only slightly narrowed. The heart muscle was distinctly fatty.

[1] Giraudeau, Sem. Med., Sept. 14, 1898. [2] Chimenti, Annual del Accad. Med. Chir. di Perugia, Vol. x. [3] Sequeira, Lancet, Dec. 31, 1898. [4] Boyd, Brit. Med. Jour., Dec. 17, 1898. [5] Herzog, Deut. Med. Woch., Nov. 10, 1898. [6] Eichhorst, Deut. Med. Woch., June 23, 1898. [7] Laache, Deut. Zeitung, Nov., 1898. [8] Short, Practitioner, Dec., 1898.

